# Penicillin allergy de-labelling implementation intervention in a UK hospital: a process evaluation, the patient experience

**DOI:** 10.1093/jacamr/dlaf144

**Published:** 2025-08-13

**Authors:** Neil Powell, Mathew Upton, Bridie Kent, Jonathan A T Sandoe, Sarah Tonkin-Crine

**Affiliations:** Pharmacy Department, Royal Cornwall Hospital, Truro TR1 3LJ, UK; School of Biomedical Sciences, University of Plymouth, Plymouth PL4 8AA, UK; School of Biomedical Sciences, University of Plymouth, Plymouth, UK; School of Nursing and Midwifery, University of Plymouth, Plymouth, UK; Leeds Institute of Medical Research, University of Leeds, Leeds, UK; Nuffield Department of Primary Care Health Sciences, University of Oxford, Oxford, UK

## Abstract

**Background:**

Penicillin allergy (penA) records are common, but true penA is rare. PenA records are associated with broad spectrum antibiotic prescribing and negative patient outcomes. We developed a behavioural intervention package to support inpatient penicillin allergy de-labelling (PADL) delivered by a multi-profession non-allergist workforce to remove incorrect penA records from medical and surgical adult inpatients in a UK hospital.

**Aims:**

To explore the experiences, beliefs and concerns of patients who had been offered PADL.

**Methods:**

Semi-structured interviews to explore the views of patients admitted to a medical or surgical ward with a penA record and offered PADL between June 2024 and October 2024. Inductive reflexive thematic analysis was used to analyse the data.

**Results:**

Twenty patients were interviewed. Patients that believed their penA to be incorrect and those that described their index reaction as mild were more likely to agree to testing. Patients considered hospital a safe place to be tested. Some patients thought being acutely unwell was not a barrier to testing, whereas others preferred an outpatient setting once discharged from hospital. De-labelled patients described having a good explanation of the risks and benefits of PADL, were grateful for the opportunity and trusted the healthcare worker and the PADL process.

**Conclusion:**

PADL was well accepted by patients who described receiving a good explanation of the PADL process. Index reactions perceived as low severity (e.g. non-severe rashes) and/or doubtful of their penA (e.g. unaware they had a penA record) were more likely to accept PADL. Some who declined inpatient PADL would consider outpatient testing once recovered from their acute illness.

## Introduction

Penicillin allergy (penA) records are common, affecting 15% of hospitalized patients, but true allergy is rare.^1,2^ PenA records are associated with broad spectrum antibiotic use, which negatively affects patients, healthcare systems and wider society.^3^ Removing incorrect penA records, termed penicillin allergy de-labelling (PADL), enabling patients to receive first line antibiotics is safe and effective.^4,5^

We developed a behavioural intervention package to support inpatient PADL delivered by a multi-profession non-allergist workforce to remove incorrect penA records from medical and surgical adult inpatients.^6^

This study explored the experiences, beliefs and concerns of patients who had been offered PADL as part of this complex intervention, launched in June 2024, and to use the findings to optimize the implementation of PADL.

## Methods

### Study design and setting

The setting was a 760-bed hospital serving a population of 450 000 people in rural UK with ∼120 000 hospital admissions per year.

Semi-structured interviews were used to explore the views of patients who had a penA record when admitted to an acute medical or surgical ward between June 2024 and October 2024 and offered PADL.

### Implementation intervention

The PADL pathway is described elsewhere, but in summary, comprised a penA focused history, risk assessment and, if low risk for a genuine allergy, counselled on risks and benefits of PADL before being offered de-labelling either on history alone or, after a test dose of a penicillin antibiotic.^6^ This was undertaken by ward clinical teams (doctor, ward pharmacist or medicines optimization pharmacy technician) or antimicrobial stewardship pharmacist. The ward nurse administered the test and if there were no symptoms within 1 hour the patient was de-labelled.

### Participant selection

We used purposive sampling to ensure representation from the following groups: (i) suitable for direct oral challenge (DOC) or direct de-label (DDL) but declined testing, (ii) suitable for DOC and accepted testing, and (iii) suitable for DDL and accepted de-label. These groups were identified to ensure participant experiences who had both declined PADL and accepted PADL were explored. Patients were telephoned, after discharge from the hospital, by N.P. and invited to participate in the study. The patient information leaflet explaining the purpose of the study was either emailed or posted to patients. Patients were recontacted after at least 24 hours after receipt of the patient information leaflet and an interview scheduled at a future interview date and time convenient to the patient. Patients were asked whether they had any questions about the study before proceeding with the interview questions. Owing to the homogeneous patient sample undergoing the same intervention at a single site, we aimed for a target of at least five patients from each of the three groups with a total sample size target of 15–20 participants, a number likely to reach saturation for common themes.^7^ An antimicrobial pharmacist (N.P.) identified patients who had been offered PADL during the study period and invited them by telephone to participate in a one-to-one interview. Patients who expressed interest in participating in the interview were contacted after at least 24 hours to answer any questions and to schedule an interview.

### Data collection

An interview guide was developed based on the primary research question, informed by existing literature and the Theoretical Domains Framework.^8–14^ The Theoretical Domains Framework provides a robust theoretical basis for implementation studies and was used to ensure all possible relevant influences on implementation of the PADL pathway were explored.^8^ The interviewer (N.P.) explained he wanted to hear about participants’ opinions and experiences of living with a penA record and their recent experience of having their penA challenged.

Participants were asked general questions exploring their understanding of penA and specific questions about their experience of being approached by healthcare workers (HCWs) and having their penA challenged (where relevant). Interviews were conducted over the telephone, audio recorded and transcribed verbatim by an independent transcription services company. N.P. conducted the interviews after obtaining recorded verbal consent. Data saturation was probably met given the homogeneous patient population and the absence of new identified themes early in the interview schedule.^7,15^

### Data analysis

Data collection and analysis took place concurrently. Transcripts were uploaded to NVivo 12. Inductive reflexive thematic analysis was used to analyse transcripts.^16^ N.P. familiarized himself with all transcripts before independently coding five transcripts, with participants from different sampling groups, before developing preliminary codes. These codes were then used to develop an initial coding framework that was then used to analyse the remaining transcripts. Further codes were added as new data were identified in later transcripts and the framework adapted as necessary.

### Ethics

This study was reviewed and approved by the Liverpool Central Research Ethics Committee (IRAS Project ID 299708).

## Results

### Participants

Thirty-five patients were invited to interview, of whom 15 declined and 20 were interviewed. Interviews were conducted between 14 October 2024 and 12 December 2024. Interviews lasted between 9 and 25 minutes (mean 14 minutes). Interviewee characteristics are reported in Table 1.

**Table 1.** Patient demographics of those who accepted the interview invitation and those who declined

Patient group	Total number of participants invited to interview	Number of participants interviewed	Age range (mean)	Male:female	Number of participants invited to interview but declined	Age range (mean)	Male:female
DDL	11	6	49–75 (60)	1:5	5	39–75 (59)	0:5
DOC	7	6	52–85 (66)	2:4	1	56	0:1
DOC/DDL decline	17	8	34–80 (69)	4:4	9	24–90 (68)	0:9
overall	35	20	34–85 (65)	7:13	15	24–90 (64)	0:15

Two themes captured the views of patients on living with a penA record and of their experiences of having this challenged in hospital.

### Patient beliefs and experiences of living with a penA record

#### Patient beliefs about whether their penA record represented genuine allergy

For the majority, the index reaction was decades ago. Several doubted whether they were genuinely penA. Participants doubted their penA for the following reasons: subsequent penicillin tolerance; a realization they may have grown out of it; no memory of the reaction; or the reaction was perceived by the patient as mild. Most of those who had considered whether their penA might not be genuine were successfully de-labelled either after DOC or by DDL.

*…because it had been such a long time and I just wondered if I really was still allergic or surely, I’d have grown out of that by now.* DOC_1

Most had not considered whether their penA was genuine or, if they had, they believed it to be genuine. Participants held their beliefs for several reasons. For one patient, their penA diagnosis was in keeping with their multiple other allergies. Others had relatives with penA records that reinforced their beliefs. Several participants remembered the reactions that had appeared very soon after starting penicillin, or got worse when taking penicillin and disappeared when they stopped the penicillin. Some said that HCWs accepting their penA status, without further exploration, reinforced their penA status for them. Just under half the participants who had either not considered their penA status or believed it to be correct had declined the offer of de-label either on history alone or after DOC.

*So it* [the rash] *came on, I think I took the penicillin in the morning and it had started in the evening*. DOC_declined_2

#### Impact of penA on past healthcare

Most said that their penA record had not negatively affected their healthcare. For some, this was because they had not needed antibiotics until their recent hospital stay. For many patients, they had required several courses of antibiotics in the past, but the antibiotics had not caused them any harm and they did not perceive them to be any less effective.

*So it* [penA record] *is of no real worry to me because they had alternative means of giving me something to make me better*. DOC declined_4

For a minority of patients, their penA record had negatively affected previous healthcare, including making it difficult to find an appropriate antibiotic for one patient and the rest reported side effects with the alternatives, such as rashes.

*I was given as an IV and my arm flared up in a huge rash. It all went red and was very itchy while they were giving me the IV of levofloxacin.* DDL_2

For nearly all patients, their penA record had not been explored by HCWs in the past. One patient had asked doctors to explore her penA because she did not believe her penA status but was declined.

*I would be quite happy in a controlled environment, i.e. the hospital, to try it, but the* [hospital] *doctors wouldn’t do it*. DDL_6

#### Beliefs about the impact of penA records on future care

Most de-labelled patients recognized the benefits of being de-labelled and were pleased they had been de-labelled. Many patients recognized that penicillin was a useful, potentially more effective, group of antibiotics. One patient said that being given access to penicillin had saved their life during their most recent hospital stay. Another said having access to penicillin made her protracted antibiotic regimen simpler and more manageable.

*So, I think certainly [amoxicillin] this instance in June, I think it saved my life really*. DDL_1

Two patients, who both declined being de-labelled, both acknowledged that PADL would provide more antibiotic options; with one acknowledging the benefit of more options given their co-morbidities already prevented the use of some antibiotics and that the alternatives are less effective but declined testing due to feeling overwhelmed with their medical situation while an inpatient.

*I think it would actually be quite helpful since I've got other medical conditions so I can’t have some of the other antibiotics so I’m quite limited*. DOC_declined_2

### Patient PADL experiences

#### Influences on agreeing to PADL

Patients who agreed to PADL said they felt safe being tested in the hospital because there was access to medical intervention should they need it, including patients who considered themselves to be very unwell. One patient, who had declined DDL while an inpatient, said if they were offered PADL again and if the exposure was under supervision, then they would reconsider taking penicillin again but did express fear with re-exposure.

*It’s good because I was surrounded with doctors, so it was good for me because I was in hospital*. DOC_3

*I was in with pneumonia, so not well at all really but I was quite willing to take the penicillin.* DOC_5

Several who had declined PADL said that while acutely unwell was not the right time to de-label patients. Patients said they were overwhelmed with all the things happening to them during their inpatient stay and that they did not feel able to think about other interventions, such as PADL. Several patients said that they would agree to testing in the outpatient setting once they had got over their acute illness. Polypharmacy made one patient reluctant to try penicillin because they were on several drugs that were not causing them problems and they did not want to upset that balance.

*So now I’m out of hospital I know it's a good idea but when I was approached in hospital I was like quite sick and dealing with so many different things*. DOC_declined_2

Three patients, all who had declined DDL, said that they did not have any recollection of being offered PADL and denied having conversations with HCWs about PADL, but they all said they would be willing to try PADL, if they were offered it.

*I don’t remember anybody asking me if I… I said I didn’t want it took off the list, or something?—I dunno. I mean, if you wanna try it again, I’d take it*. DDL_Decline 2

#### Severity and certainty of index reaction diagnosis influences decision to accept PADL

Those who agreed to PADL described their reactions as mild and were willing to take penicillin again believing that the worst they were going to experience was the same reaction again, which were described as mild rashes or thrush. One participant agreed to PADL as they were unaware of their penA record and had no recollection of taking penicillin in the past.

*…just having a little rash on the stomach I mean, it wasn’t a bad, bad like I say ’cause the other one [erythromycin], you know when my face swelled up I wouldn’t have dreamt of trying it*. DOC_5

Participants who declined PADL often described their index reactions as ‘severe’ and were unwilling to be re-exposed through fear of experiencing the same reaction. One patient described a benign skin reaction, the patient themselves was convinced it was an allergic reaction, which was supported by their GP.

*Put it this way, I’m afraid to try it again because it was quite unpleasant, it was quite painful*. DOC_decline_5

#### Understanding the risks and benefits of testing

Several participants reported receiving adequate information about the benefits of PADL. Those de-labelled by the ward teams described being given a good explanation of the benefits of PADL. Several patients de-labelled after an oral challenge said they were given an adequate explanation of the risks and several said that as well as the verbal explanation they received some written information on PADL but did acknowledge their memory of the content was poor. Those de-labelled by DDL were vaguer about the risks with several patients saying that they were not aware of the risks.

*…it was all very clearly explained to me and I was quite happy to go ahead.* DOC_7

#### Trust in the PADL process and PADL outcome

De-labelled patients said they had confidence in the PADL process, and were grateful to have been de-labelled and said they would take penicillin in the future. Patients valued the time taken by HCWs to explain the PADL process and valued the good explanations of the risks, benefits and the testing procedure. Patients said they were confident going ahead with PADL because they trusted the HCW de-labelling them and trusted that they would not come to any harm. When asked about ways to improve the PADL process participants were unable to offer suggestions they thought would improve their experience, but that PADL should be more widely available to patients.

*Yes they did, he went through it very thoroughly and made sure I was happy and told me that I’d be monitored*. DOC_7

One patient described not being monitored due to the ward staff being busy. One patient enquired in the interview about how they would get their GP record updated, which may reflect their forgetfulness of that part of the explanation or our omission to convey to the patient that we would inform their GP post de-label. Several patients talked about their forgetfulness and particularly in the context of their acute admission where they were experiencing a lot of anxiety due to the many things going on for them.

*Did they tell you about any risks to you of having it removed? Not that I remember no. No, not at all*. DDL_2

Two patients questioned whether a single dose of penicillin was enough to rule out penA. One patient, who declined DOC, was concerned that if she tolerated a single dose, was then de-labelled and then went on to receive a full course of penicillin that potentially something ‘horrendous’ might happen later. One patient who was de-labelled after a single dose was not wholly convinced and was not sure whether she should or should not be reporting her penA to HCWs.

*How can you possibly surmise that, after one hour, somebody isn’t allergic? I mean, with me, it was probably a couple of days after I started taking the tablets that these lumps came up*. DOC declined_4

## Discussion

### Main findings

We identified a relationship between severity and/or certainty of prior reaction to penicillin and participant willingness to be tested. Some participants believed their penA to be incorrect and were likely to agree to testing, whereas those that believed their penA to be genuine were more likely to decline testing. Patients who described index reactions as mild were likely to agree to PADL except for those with benign reactions but who were convinced their penA was genuine. Several patients who declined testing perceived their index reaction to be severe and feared experiencing the same reaction again.

Those who agreed to PADL considered hospital a safe place to be tested and that being acutely unwell was not a barrier to testing, whereas others felt that while acutely unwell was not the right time to be tested and described being too overwhelmed to consider PADL. Several patients who declined PADL thought the outpatient setting, when over their acute illness, was a better option and would be willing to consider PADL, highlighting the importance of the clinical context in which patients are approached. This may demonstrate that for some patients who initially decline PADL, provision of further information on the benefits of testing and reassurance of the low risk of reactions when exposed to penicillin, more time to consider PADL and offering outpatient testing might persuade more patients to agree.

Some participants who had agreed to PADL described having a good explanation of the risks and benefits of PADL and were grateful of the time HCWs had spent to explore their penA record and to remove it, while others had no recollection of this discussion. Providing written information can be a way of overcoming the problem of recollecting key information in complex clinical consultations but this idea was not welcomed by all participants. De-labelled patients trusted the HCW and the PADL process.

Most patients were unaware of the impact of penA records on their healthcare but patients who were de-labelled largely acknowledged the benefits to them of PADL for their future care, probably due to the discussion they had had with HCWs about PADL during their recent inpatient stay. Wider patient education and awareness raising of the benefits and risks of PADL may increase patient knowledge and awareness of PADL and in turn motivate more patients to agree to PADL while an inpatient.

### Comparison with the literature

Ngassa *et al.* explored US patients’ views on PADL and found that most people interviewed were interested in PADL, even those reporting a previous severe reaction to penicillin.^9^ There was no clear connection between severity of index reaction and willingness to agree to PADL, although negative memories of receiving penicillin made patients more cautious about PADL, contrary to our findings and those from Santillo *et al.* who also found that patients who had agreed to PADL had doubted their penA record before agreeing to testing.^17^

There were several similarities with the study by Augustino *et al*. that found a minority of their US patients were aware of the impact of avoiding penicillin on their healthcare, which was also a finding in a UK study.^11,13^ Some patients expressed fear of a bad reaction during the PADL process but said that a good explanation of the PADL process reduced patient concerns, and nearly all patients reported satisfaction with the PADL process and reported confidence in a person delivering PADL who communicated well.^13^ Similarly, those de-labelled were pleased to no longer be labelled as allergic to penicillin and most were confident in the negative result.^13^ However, there were some concerns from patients that their negative test may be a false negative, but most would recommend PADL to others.^13^ Santillo *et al.* reported similar findings with patients stating they were well informed about the testing process and reassured by personable and approachable staff, and that they felt safe and trusted the testing process.^17^

Ngassa *et al.* and Jani *et al.* both found many US and UK patients did not see a reason for testing due to there being several other effective antibiotic treatment options available.^13,14^ Similar to our study, Jani *et al.* report poor health as a reason given by UK patients for not agreeing to DOC, and that patients were reassured by PADL conducted in the hospital setting had confidence in the staff involved in their PADL with all those undergoing DOC reporting a positive experience.^14^ Wilson *et al.* explored the views of their Australian inpatients undergoing PADL and found that nearly all patients felt both safe and would recommend PADL to others.^12^

### Strengths and limitations

Although our study was limited by recruitment from a single centre, many findings align well with previously published work. We recruited in a predominantly white population with all interviewed participants being white, so the findings may not be applicable in non-white ethnic groups.^18^ We did not explore socio-economic deprivation within the interviewed patients, which further limits our findings.

We could explore the views of both those that accepted and declined PADL and elicit views across a range of the patient population. The views of our patients are comparable to those of PADL patients in another UK study and studies from the USA and Australia.^9,12–14^

### Implications for practice and research

Our findings highlight how implementation interventions need to be detailed enough to target specific influences on patient behaviours. Public awareness raising and education campaigns about the high rates of incorrect penicillin allergy records and benefits of PADL may encourage more patients to consider whether their penicillin allergy record is genuine before any offer of testing, which may increase PADL uptake. The public awareness messaging and key educational components that would encourage patients to consider their circumstances and encourage them to accept testing, when offered, needs further exploration with patients through qualitative research.

HCWs and the patient information materials need to reassure patients that a test using one dose of penicillin is a trustworthy and a thorough testing process, and that PADL is being carried out in a safe place with access to medical intervention, should it be needed. Patients value a good explanation of risks and benefits of PADL, a good explanation of the PADL process and they value the time taken for this. Having confidence and trust in the person providing information increases patient acceptance of PADL.

Some patients have no recollection of being offered PADL but suggested during the interview that they would accept PADL if offered again. Revisiting patients who initially decline testing may increase PADL uptake. Likewise offering an outpatient PADL service for those who decline due to illness acuity could also increase PADL uptake. How best to deliver low risk PADL outpatient clinics in the NHS requires further research.

### Conclusions

Being offered PADL while acutely unwell was acceptable to many patients who described receiving a good explanation of the PADL process and were grateful for the opportunity to be de-labelled. Index reactions that patients perceived as low severity and/or doubted their penA was genuine were more likely to accept PADL. Some of those who declined inpatient PADL would consider outpatient testing when recovered from their acute illness.
